# Supplementary material for: Transcriptome Analysis Reveals Vimentin-Induced Disruption of Cell–Cell Associations Augments Breast Cancer Cell Migration
Source: Cells. 2022 Dec 13;11(24):4035. doi: 10.3390/cells11244035 (PMC9776984; doi:10.3390/cells11244035)
Supplement: Supplementary file 1 [file cells-11-04035-s001.zip › DEGs and enrichment analysis.zip!/DEGs and enrichment analysis/WT_VIMvsVector GO enrichment/GSEA/WT_VIM/INTERMEDIATE_FILAMENT/INTERMEDIATE_FILAMENT(GO_0005882).html]

Details for gene set INTERMEDIATE\_FILAMENT(GO:0005882)[GSEA]


|  || Dataset | fpkm.sample |
| Phenotype | sample.cls |
| Upregulated in class | WT\_VIM |
| GeneSet | INTERMEDIATE\_FILAMENT(GO:0005882) |
| Enrichment Score (ES) | 0.90280485 |
| Normalized Enrichment Score (NES) | 1.4616249 |
| Nominal p-value | 0.0 |
| FDR q-value | 0.16300032 |
| FWER p-Value | 0.163 |
Table: GSEA Results Summary

  

Fig 1: Enrichment plot: INTERMEDIATE\_FILAMENT(GO:0005882)
  
Profile of the Running ES Score & Positions of GeneSet Members on the Rank Ordered List

  

| PROBE | DESCRIPTION   (from dataset) | GENE SYMBOL | GENE\_TITLE | RANK IN GENE LIST | RANK METRIC SCORE | RUNNING ES | CORE ENRICHMENT || 1 | ENSG00000255275 | AL080251.1 |  |  | 14 | Infinity | -0.0002 | Yes |
| 2 | ENSG00000189182 | KRT77 |  |  | 1537 | Infinity | -0.0261 | Yes |
| 3 | ENSG00000186442 | KRT3 |  |  | 1666 | Infinity | -0.0282 | Yes |
| 4 | ENSG00000197079 | KRT35 |  |  | 2099 | Infinity | -0.0355 | Yes |
| 5 | ENSG00000175084 | DES |  |  | 2208 | Infinity | -0.0374 | Yes |
| 6 | ENSG00000167768 | KRT1 |  |  | 2257 | Infinity | -0.0382 | Yes |
| 7 | ENSG00000026025 | VIM |  |  | 2388 | 1709.539 | 0.8840 | Yes |
| 8 | ENSG00000164850 | GPER1 |  |  | 2450 | 15.517 | 0.8914 | Yes |
| 9 | ENSG00000108759 | KRT32 |  |  | 2494 | 11.542 | 0.8969 | Yes |
| 10 | ENSG00000171401 | KRT13 |  |  | 2640 | 7.435 | 0.8984 | Yes |
| 11 | ENSG00000132688 | NES |  |  | 2798 | 5.994 | 0.8990 | Yes |
| 12 | ENSG00000185069 | KRT76 |  |  | 2810 | 5.946 | 0.9020 | Yes |
| 13 | ENSG00000167916 | KRT24 |  |  | 2933 | 5.245 | 0.9028 | Yes |
| 14 | ENSG00000007001 | UPP2 |  |  | 3092 | 4.618 | 0.9026 | No |
| 15 | ENSG00000204889 | KRT40 |  |  | 3311 | 3.983 | 0.9011 | No |
| 16 | ENSG00000138587 | MNS1 |  |  | 4247 | 2.821 | 0.8867 | No |
| 17 | ENSG00000176619 | LMNB2 |  |  | 4590 | 2.621 | 0.8824 | No |
| 18 | ENSG00000113368 | LMNB1 |  |  | 4598 | 2.616 | 0.8836 | No |
| 19 | ENSG00000081277 | PKP1 |  |  | 4663 | 2.579 | 0.8840 | No |
| 20 | ENSG00000135406 | PRPH |  |  | 5036 | 2.385 | 0.8789 | No |
| 21 | ENSG00000239672 | NME1 |  |  | 5076 | 2.363 | 0.8795 | No |
| 22 | ENSG00000170442 | KRT86 |  |  | 5441 | 2.218 | 0.8746 | No |
| 23 | ENSG00000213672 | NCKIPSD |  |  | 5937 | 2.050 | 0.8673 | No |
| 24 | ENSG00000160789 | LMNA |  |  | 5996 | 2.035 | 0.8674 | No |
| 25 | ENSG00000170819 | BFSP2 |  |  | 6181 | 1.992 | 0.8654 | No |
| 26 | ENSG00000185479 | KRT6B |  |  | 6472 | 1.931 | 0.8615 | No |
| 27 | ENSG00000100285 | NEFH |  |  | 6771 | 1.860 | 0.8574 | No |
| 28 | ENSG00000162520 | SYNC |  |  | 7674 | 1.682 | 0.8430 | No |
| 29 | ENSG00000171403 | KRT9 |  |  | 7795 | 1.660 | 0.8419 | No |
| 30 | ENSG00000171345 | KRT19 |  |  | 7870 | 1.645 | 0.8415 | No |
| 31 | ENSG00000180921 | FAM83H |  |  | 7944 | 1.632 | 0.8412 | No |
| 32 | ENSG00000186081 | KRT5 |  |  | 8964 | 1.487 | 0.8247 | No |
| 33 | ENSG00000188878 | FBF1 |  |  | 9031 | 1.476 | 0.8244 | No |
| 34 | ENSG00000170421 | KRT8 |  |  | 9203 | 1.452 | 0.8222 | No |
| 35 | ENSG00000244411 | KRTAP5-7 |  |  | 9325 | 1.439 | 0.8210 | No |
| 36 | ENSG00000170523 | KRT83 |  |  | 10024 | 1.356 | 0.8099 | No |
| 37 | ENSG00000111057 | KRT18 |  |  | 10205 | 1.334 | 0.8075 | No |
| 38 | ENSG00000186847 | KRT14 |  |  | 11204 | 1.247 | 0.7913 | No |
| 39 | ENSG00000173801 | JUP |  |  | 11282 | 1.238 | 0.7906 | No |
| 40 | ENSG00000161849 | KRT84 |  |  | 11438 | 1.230 | 0.7887 | No |
| 41 | ENSG00000135480 | KRT7 |  |  | 12155 | 1.169 | 0.7771 | No |
| 42 | ENSG00000057294 | PKP2 |  |  | 12177 | 1.167 | 0.7774 | No |
| 43 | ENSG00000171346 | KRT15 |  |  | 12263 | 1.159 | 0.7766 | No |
| 44 | ENSG00000182253 | SYNM |  |  | 13149 | 1.091 | 0.7622 | No |
| 45 | ENSG00000204572 | KRTAP5-10 |  |  | 13171 | 1.090 | 0.7624 | No |
| 46 | ENSG00000115902 | SLC1A4 |  |  | 13387 | 1.073 | 0.7593 | No |
| 47 | ENSG00000139437 | TCHP |  |  | 13407 | 1.071 | 0.7596 | No |
| 48 | ENSG00000141562 | NARF |  |  | 13691 | 1.050 | 0.7554 | No |
| 49 | ENSG00000131095 | GFAP |  |  | 14245 | 1.007 | 0.7465 | No |
| 50 | ENSG00000157978 | LDLRAP1 |  |  | 14321 | 1.001 | 0.7458 | No |
| 51 | ENSG00000128271 | ADORA2A |  |  | 14382 | 0.996 | 0.7453 | No |
| 52 | ENSG00000198947 | DMD |  |  | 14471 | 0.996 | 0.7444 | No |
| 53 | ENSG00000170465 | KRT6C |  |  | 14562 | 0.996 | 0.7434 | No |
| 54 | ENSG00000198443 | KRTAP4-1 |  |  | 14696 | 0.989 | 0.7417 | No |
| 55 | ENSG00000135443 | KRT85 |  |  | 15094 | 0.964 | 0.7354 | No |
| 56 | ENSG00000186832 | KRT16 |  |  | 15363 | 0.949 | 0.7314 | No |
| 57 | ENSG00000205426 | KRT81 |  |  | 16270 | 0.901 | 0.7165 | No |
| 58 | ENSG00000143631 | FLG |  |  | 16655 | 0.879 | 0.7105 | No |
| 59 | ENSG00000100941 | PNN |  |  | 17222 | 0.843 | 0.7013 | No |
| 60 | ENSG00000254997 | KRTAP5-9 |  |  | 17318 | 0.838 | 0.7002 | No |
| 61 | ENSG00000186395 | KRT10 |  |  | 18257 | 0.784 | 0.6847 | No |
| 62 | ENSG00000146872 | TLK2 |  |  | 18549 | 0.769 | 0.6802 | No |
| 63 | ENSG00000162105 | SHANK2 |  |  | 18761 | 0.756 | 0.6770 | No |
| 64 | ENSG00000128881 | TTBK2 |  |  | 19042 | 0.740 | 0.6726 | No |
| 65 | ENSG00000096696 | DSP |  |  | 19375 | 0.721 | 0.6674 | No |
| 66 | ENSG00000113712 | CSNK1A1 |  |  | 19674 | 0.706 | 0.6627 | No |
| 67 | ENSG00000197535 | MYO5A |  |  | 20877 | 0.642 | 0.6427 | No |
| 68 | ENSG00000241233 | KRTAP5-8 |  |  | 21129 | 0.629 | 0.6388 | No |
| 69 | ENSG00000125864 | BFSP1 |  |  | 21341 | 0.617 | 0.6355 | No |
| 70 | ENSG00000205420 | KRT6A |  |  | 21403 | 0.615 | 0.6348 | No |
| 71 | ENSG00000099250 | NRP1 |  |  | 22101 | 0.571 | 0.6233 | No |
| 72 | ENSG00000105141 | CASP14 |  |  | 22288 | 0.558 | 0.6204 | No |
| 73 | ENSG00000135596 | MICAL1 |  |  | 22417 | 0.550 | 0.6186 | No |
| 74 | ENSG00000167767 | KRT80 |  |  | 22813 | 0.520 | 0.6121 | No |
| 75 | ENSG00000151914 | DST |  |  | 23103 | 0.498 | 0.6075 | No |
| 76 | ENSG00000130779 | CLIP1 |  |  | 24939 | 0.360 | 0.5766 | No |
| 77 | ENSG00000010295 | IFFO1 |  |  | 25048 | 0.347 | 0.5749 | No |
| 78 | ENSG00000128422 | KRT17 |  |  | 25113 | 0.336 | 0.5740 | No |
| 79 | ENSG00000152936 | LMNTD1 |  |  | 25737 | 0.278 | 0.5636 | No |
| 80 | ENSG00000152661 | GJA1 |  |  | 25762 | 0.276 | 0.5633 | No |
| 81 | ENSG00000169991 | IFFO2 |  |  | 25961 | 0.249 | 0.5601 | No |
| 82 | ENSG00000204571 | KRTAP5-11 |  |  | 25975 | 0.249 | 0.5600 | No |
| 83 | ENSG00000170477 | KRT4 |  |  | 27211 | 0.051 | 0.5391 | No |
| 84 | ENSG00000187026 | KRTAP21-2 |  |  | 27334 | 0.000 | 0.5370 | No |
| 85 | ENSG00000170486 | KRT72 |  |  | 27668 | 0.000 | 0.5314 | No |
| 86 | ENSG00000170484 | KRT74 |  |  | 27669 | 0.000 | 0.5314 | No |
| 87 | ENSG00000161850 | KRT82 |  |  | 28245 | 0.000 | 0.5216 | No |
| 88 | ENSG00000172867 | KRT2 |  |  | 29036 | 0.000 | 0.5082 | No |
| 89 | ENSG00000213424 | KRT222 |  |  | 29764 | 0.000 | 0.4959 | No |
| 90 | ENSG00000185640 | KRT79 |  |  | 30570 | 0.000 | 0.4822 | No |
| 91 | ENSG00000170454 | KRT75 |  |  | 30709 | 0.000 | 0.4799 | No |
| 92 | ENSG00000094796 | KRT31 |  |  | 30994 | NaN | 0.4751 | No |
| 93 | ENSG00000212658 | KRTAP29-1 |  |  | 31147 | NaN | 0.4725 | No |
| 94 | ENSG00000212657 | KRTAP16-1 |  |  | 31150 | NaN | 0.4725 | No |
| 95 | ENSG00000186860 | KRTAP17-1 |  |  | 31817 | NaN | 0.4612 | No |
| 96 | ENSG00000241598 | KRTAP5-4 |  |  | 32370 | NaN | 0.4518 | No |
| 97 | ENSG00000185940 | KRTAP5-5 |  |  | 32555 | NaN | 0.4487 | No |
| 98 | ENSG00000196156 | KRTAP4-3 |  |  | 32637 | NaN | 0.4473 | No |
| 99 | ENSG00000198083 | KRTAP9-9 |  |  | 32652 | NaN | 0.4471 | No |
| 100 | ENSG00000198390 | KRTAP13-1 |  |  | 32692 | NaN | 0.4464 | No |
| 101 | ENSG00000212659 | KRTAP9-6 |  |  | 32796 | NaN | 0.4447 | No |
| 102 | ENSG00000212899 | KRTAP3-3 |  |  | 32883 | NaN | 0.4432 | No |
| 103 | ENSG00000184351 | KRTAP19-1 |  |  | 33199 | NaN | 0.4379 | No |
| 104 | ENSG00000139648 | KRT71 |  |  | 33878 | NaN | 0.4264 | No |
| 105 | ENSG00000126337 | KRT36 |  |  | 34009 | NaN | 0.4242 | No |
| 106 | ENSG00000196224 | KRTAP5-3 |  |  | 34593 | NaN | 0.4143 | No |
| 107 | ENSG00000197683 | KRTAP26-1 |  |  | 34645 | NaN | 0.4135 | No |
| 108 | ENSG00000171446 | KRT27 |  |  | 34747 | NaN | 0.4117 | No |
| 109 | ENSG00000171396 | KRTAP4-4 |  |  | 35211 | NaN | 0.4039 | No |
| 110 | ENSG00000006059 | KRT33A |  |  | 36194 | NaN | 0.3872 | No |
| 111 | ENSG00000148798 | INA |  |  | 37591 | NaN | 0.3636 | No |
| 112 | ENSG00000186971 | KRTAP13-4 |  |  | 37686 | NaN | 0.3620 | No |
| 113 | ENSG00000186977 | KRTAP19-5 |  |  | 37687 | NaN | 0.3620 | No |
| 114 | ENSG00000108417 | KRT37 |  |  | 37697 | NaN | 0.3618 | No |
| 115 | ENSG00000272804 | KRTAP10-7 |  |  | 37881 | NaN | 0.3587 | No |
| 116 | ENSG00000215455 | KRTAP10-1 |  |  | 37917 | NaN | 0.3581 | No |
| 117 | ENSG00000182816 | KRTAP13-2 |  |  | 37988 | NaN | 0.3570 | No |
| 118 | ENSG00000180386 | KRTAP9-7 |  |  | 38752 | NaN | 0.3440 | No |
| 119 | ENSG00000198271 | KRTAP4-5 |  |  | 39977 | NaN | 0.3233 | No |
| 120 | ENSG00000188155 | KRTAP10-6 |  |  | 40395 | NaN | 0.3162 | No |
| 121 | ENSG00000186924 | KRTAP22-1 |  |  | 40752 | NaN | 0.3101 | No |
| 122 | ENSG00000186925 | KRTAP19-6 |  |  | 40753 | NaN | 0.3102 | No |
| 123 | ENSG00000182591 | KRTAP11-1 |  |  | 40849 | NaN | 0.3085 | No |
| 124 | ENSG00000213417 | KRTAP2-4 |  |  | 41124 | NaN | 0.3039 | No |
| 125 | ENSG00000189169 | KRTAP10-12 |  |  | 41179 | NaN | 0.3030 | No |
| 126 | ENSG00000206107 | KRTAP27-1 |  |  | 41184 | NaN | 0.3029 | No |
| 127 | ENSG00000206106 | KRTAP22-2 |  |  | 41185 | NaN | 0.3029 | No |
| 128 | ENSG00000206105 | KRTAP20-4 |  |  | 41186 | NaN | 0.3029 | No |
| 129 | ENSG00000206102 | KRTAP19-8 |  |  | 41189 | NaN | 0.3029 | No |
| 130 | ENSG00000241241 | KRTAP4-16 |  |  | 41352 | NaN | 0.3002 | No |
| 131 | ENSG00000241123 | KRTAP10-5 |  |  | 41457 | NaN | 0.2984 | No |
| 132 | ENSG00000183640 | KRTAP8-1 |  |  | 41820 | NaN | 0.2923 | No |
| 133 | ENSG00000212938 | KRTAP6-3 |  |  | 41896 | NaN | 0.2910 | No |
| 134 | ENSG00000206104 | KRTAP20-3 |  |  | 42199 | NaN | 0.2859 | No |
| 135 | ENSG00000241595 | KRTAP9-4 |  |  | 42281 | NaN | 0.2845 | No |
| 136 | ENSG00000188694 | KRTAP24-1 |  |  | 42794 | NaN | 0.2758 | No |
| 137 | ENSG00000212722 | KRTAP4-9 |  |  | 42843 | NaN | 0.2750 | No |
| 138 | ENSG00000212721 | KRTAP4-11 |  |  | 42844 | NaN | 0.2750 | No |
| 139 | ENSG00000212724 | KRTAP2-3 |  |  | 42845 | NaN | 0.2750 | No |
| 140 | ENSG00000212725 | KRTAP2-1 |  |  | 42846 | NaN | 0.2750 | No |
| 141 | ENSG00000108244 | KRT23 |  |  | 42848 | NaN | 0.2750 | No |
| 142 | ENSG00000198090 | KRTAP4-6 |  |  | 43033 | NaN | 0.2719 | No |
| 143 | ENSG00000244362 | KRTAP19-7 |  |  | 43626 | NaN | 0.2619 | No |
| 144 | ENSG00000244537 | KRTAP4-2 |  |  | 44049 | NaN | 0.2547 | No |
| 145 | ENSG00000204887 | KRTAP1-4 |  |  | 44485 | NaN | 0.2473 | No |
| 146 | ENSG00000204880 | KRTAP4-8 |  |  | 44486 | NaN | 0.2473 | No |
| 147 | ENSG00000186393 | KRT26 |  |  | 44569 | NaN | 0.2460 | No |
| 148 | ENSG00000186049 | KRT73 |  |  | 45092 | NaN | 0.2371 | No |
| 149 | ENSG00000244624 | KRTAP20-1 |  |  | 45823 | NaN | 0.2247 | No |
| 150 | ENSG00000244025 | KRTAP19-3 |  |  | 45865 | NaN | 0.2240 | No |
| 151 | ENSG00000215454 | KRTAP10-4 |  |  | 45887 | NaN | 0.2237 | No |
| 152 | ENSG00000214518 | KRTAP2-2 |  |  | 46182 | NaN | 0.2187 | No |
| 153 | ENSG00000274749 | KRTAP7-1 |  |  | 46377 | NaN | 0.2154 | No |
| 154 | ENSG00000171360 | KRT38 |  |  | 46626 | NaN | 0.2112 | No |
| 155 | ENSG00000231068 | KRTAP21-3 |  |  | 47053 | NaN | 0.2040 | No |
| 156 | ENSG00000186980 | KRTAP23-1 |  |  | 47265 | NaN | 0.2004 | No |
| 157 | ENSG00000205864 | KRTAP5-6 |  |  | 47576 | NaN | 0.1952 | No |
| 158 | ENSG00000205867 | KRTAP5-2 |  |  | 47577 | NaN | 0.1952 | No |
| 159 | ENSG00000205869 | KRTAP5-1 |  |  | 47578 | NaN | 0.1952 | No |
| 160 | ENSG00000204873 | KRTAP9-3 |  |  | 47721 | NaN | 0.1928 | No |
| 161 | ENSG00000239886 | KRTAP9-2 |  |  | 48257 | NaN | 0.1837 | No |
| 162 | ENSG00000196859 | KRT39 |  |  | 48263 | NaN | 0.1836 | No |
| 163 | ENSG00000232263 | KRTAP25-1 |  |  | 48579 | NaN | 0.1783 | No |
| 164 | ENSG00000205445 | KRTAP10-2 |  |  | 49041 | NaN | 0.1705 | No |
| 165 | ENSG00000240871 | KRTAP4-7 |  |  | 49061 | NaN | 0.1701 | No |
| 166 | ENSG00000188581 | KRTAP1-1 |  |  | 49262 | NaN | 0.1668 | No |
| 167 | ENSG00000213416 | KRTAP4-12 |  |  | 49268 | NaN | 0.1667 | No |
| 168 | ENSG00000221880 | KRTAP1-3 |  |  | 49483 | NaN | 0.1631 | No |
| 169 | ENSG00000187242 | KRT12 |  |  | 49652 | NaN | 0.1602 | No |
| 170 | ENSG00000186970 | KRTAP15-1 |  |  | 49904 | NaN | 0.1560 | No |
| 171 | ENSG00000240542 | KRTAP9-1 |  |  | 50174 | NaN | 0.1514 | No |
| 172 | ENSG00000104722 | NEFM |  |  | 50333 | NaN | 0.1487 | No |
| 173 | ENSG00000240432 | KRTAP13-3 |  |  | 51208 | NaN | 0.1339 | No |
| 174 | ENSG00000131737 | KRT34 |  |  | 51305 | NaN | 0.1323 | No |
| 175 | ENSG00000131738 | KRT33B |  |  | 51308 | NaN | 0.1322 | No |
| 176 | ENSG00000186930 | KRTAP6-2 |  |  | 51485 | NaN | 0.1293 | No |
| 177 | ENSG00000221852 | KRTAP1-5 |  |  | 51751 | NaN | 0.1248 | No |
| 178 | ENSG00000221859 | KRTAP10-10 |  |  | 51755 | NaN | 0.1247 | No |
| 179 | ENSG00000184724 | KRTAP6-1 |  |  | 51861 | NaN | 0.1230 | No |
| 180 | ENSG00000173908 | KRT28 |  |  | 52236 | NaN | 0.1166 | No |
| 181 | ENSG00000184032 | KRTAP20-2 |  |  | 52621 | NaN | 0.1101 | No |
| 182 | ENSG00000187272 | KRTAP9-8 |  |  | 52828 | NaN | 0.1066 | No |
| 183 | ENSG00000212900 | KRTAP3-2 |  |  | 53069 | NaN | 0.1025 | No |
| 184 | ENSG00000212901 | KRTAP3-1 |  |  | 53070 | NaN | 0.1025 | No |
| 185 | ENSG00000187766 | KRTAP10-8 |  |  | 53822 | NaN | 0.0898 | No |
| 186 | ENSG00000170423 | KRT78 |  |  | 54026 | NaN | 0.0864 | No |
| 187 | ENSG00000221864 | KRTAP12-2 |  |  | 54891 | NaN | 0.0717 | No |
| 188 | ENSG00000204897 | KRT25 |  |  | 55079 | NaN | 0.0686 | No |
| 189 | ENSG00000187175 | KRTAP12-1 |  |  | 55219 | NaN | 0.0662 | No |
| 190 | ENSG00000186965 | KRTAP19-2 |  |  | 55682 | NaN | 0.0584 | No |
| 191 | ENSG00000186967 | KRTAP19-4 |  |  | 55683 | NaN | 0.0584 | No |
| 192 | ENSG00000187005 | KRTAP21-1 |  |  | 55689 | NaN | 0.0583 | No |
| 193 | ENSG00000198010 | DLGAP2 |  |  | 55733 | NaN | 0.0576 | No |
| 194 | ENSG00000243489 | KRTAP10-11 |  |  | 56183 | NaN | 0.0500 | No |
| 195 | ENSG00000277586 | NEFL |  |  | 56226 | NaN | 0.0492 | No |
| 196 | ENSG00000212935 | KRTAP10-3 |  |  | 56367 | NaN | 0.0469 | No |
| 197 | ENSG00000212933 | KRTAP12-4 |  |  | 56368 | NaN | 0.0469 | No |
| 198 | ENSG00000205439 | KRTAP12-3 |  |  | 58261 | NaN | 0.0148 | No |
| 199 | ENSG00000171431 | KRT20 |  |  | 59046 | NaN | 0.0015 | No |
| 200 | ENSG00000221837 | KRTAP10-9 |  |  | 59093 | NaN | 0.0007 | No |
Table: GSEA details
[plain text format]

  

Fig 2: INTERMEDIATE\_FILAMENT(GO:0005882)
  
Blue-Pink O' Gram in the Space of the Analyzed GeneSet

  

Fig 3: INTERMEDIATE\_FILAMENT(GO:0005882): Random ES distribution
  
Gene set null distribution of ES for
 **INTERMEDIATE\_FILAMENT(GO:0005882)**
